# Potential Role of Extracellular ATP Released by Bacteria in Bladder Infection and Contractility

**DOI:** 10.1128/mSphere.00439-19

**Published:** 2019-09-04

**Authors:** Behnam Abbasian, Aidan Shair, David B. O’Gorman, Ana M. Pena-Diaz, Liam Brennan, Kathleen Engelbrecht, David W. Koenig, Gregor Reid, Jeremy P. Burton

**Affiliations:** a Department of Microbiology and Immunology, The University of Western Ontario, London, Ontario, Canada; b Lawson Health Research Institute, St. Joseph’s Hospital, London, Ontario, Canada; c Plastic and Reconstructive Surgery, Department of Surgery, The University of Western Ontario, London, Ontario, Canada; d Division of Urology, Department of Surgery, The University of Western Ontario, London, Ontario, Canada; e Kimberly Clark Corporation, Global Research & Engineering, Neenah, Wisconsin, USA; University of California, Davis

**Keywords:** ATP, *Escherichia coli*, *Gardnerella*, *Lactobacillus*, extracellular

## Abstract

Urgency urinary incontinence (UUI) and overactive bladder (OAB) can both potentially be influenced by commensal and urinary tract infection-associated bacteria. The sensing of bladder filling involves interplay between various components of the nervous system, eventually resulting in contraction of the detrusor muscle during micturition. This study models host responses to various urogenital bacteria, first by using urothelial bladder cell lines and then with myofibroblast contraction assays. To measure responses, we examined Ca^2+^ influx, gene expression, and alpha smooth muscle actin deposition assays. Organisms such as Escherichia coli and Gardnerella vaginalis were found to strongly induce Ca^2+^ influx and contraction, whereas Lactobacillus crispatus and L. gasseri did not induce this response. Additionally, supernatants from lactobacilli impeded Ca^2+^ influx and contraction induced by uropathogens. Upon further investigation of factors associated with purinergic signaling pathways, the Ca^2+^ influx and contraction of cells correlated with the amount of extracellular ATP produced by E. coli. Certain lactobacilli appear to mitigate this response by utilizing extracellular ATP or producing inhibitory compounds that may act as a receptor agonist or Ca^2+^ channel blocker. These findings suggest that members of the urinary microbiota may be influencing UUI or OAB.

**IMPORTANCE** The ability of uropathogenic bacteria to release excitatory compounds, such as ATP, may act as a virulence factor to stimulate signaling pathways that could have profound effects on the urothelium, perhaps extending to the vagina. This may be countered by the ability of certain commensal urinary microbiota constituents, such as lactobacilli. Further understanding of these interactions is important for the treatment and prevention of UUI and OAB. The clinical implications may require a more targeted approach to enhance the commensal bacteria and reduce ATP release by pathogens.

## Abstract (precis)

The ability of uropathogenic bacteria to release excitatory compounds, such as ATP, may act as a virulence factor to stimulate signaling pathways that could have profound effects on the urothelium, perhaps extending to the vagina. This may be countered by the ability of certain commensal urinary microbiota constituents, such as lactobacilli. Further understanding of these interactions is important for the treatment and prevention of UUI and OAB. The clinical implications may require a more targeted approach to enhance the commensal bacteria and reduce ATP release by pathogens.

## INTRODUCTION

Urinary incontinence is common in women, but is underreported and undertreated (1, 2). Patients who suffer from overactive bladder syndrome (OAB) or urgency urinary incontinence (UUI) usually experience the sensation to urinate whether the bladder is full or not. While there are many factors involved, ultimately it is the contraction of bladder smooth muscle cells that invokes urination (2–4). The storage and voiding of urine are controlled by both the sympathetic and parasympathetic nervous system pathways (2–4). It is speculated that neurotransmitters with different effects and potentially originating from bacteria may play major roles in bladder function (5–7).

The discovery of urinary microbiota has shown that diversity differs between healthy people and patients with neurogenic bladder dysfunction, interstitial cystitis, UUI, and sexually transmitted infections (8–15). The microbial diversity in women with UUI may be associated with severity of the condition (14, 16). The genus *Lactobacillus* has been found more frequently in healthy subjects than patients with UUI (60% versus 43%), while *Gardnerella* was more abundant in patients (26% versus 12% in controls) (9). Interestingly, in one study, Lactobacillus gasseri was considerably more prevalent in UUI patients than Lactobacillus crispatus (14).

It may seem difficult to envisage how the detrusor muscle, which controls micturition, could be affected by bacteria present at the urothelial layer. Yet, the urothelium is only 3 to 5 mm thick, and uropathogens have been shown to damage and invade this layer (3). Urothelial cells communicate with the suburethral tissue in the lamina propria, which contains nerve fibers and smooth muscle cells, by releasing excitatory compounds such as ATP (3, 4). Bacterial compounds could induce urothelial cells to release excitatory compounds into the suburethral space, thereby inducing smooth muscle contraction and voiding (17–19). The hypothesis of this study is that bacteria produce, release, and potentially sequester excitatory compounds that may play a role in UUI pathogenesis. A corollary is that commensal bacteria may be beneficial by preventing detrusor muscle contractions.

This study explores interactions of uropathogenic bacteria and commensal lactobacilli to affect the physiology of bladder cells in culture and to release ATP to stimulate Ca^2+^ influx and contraction of myofibroblasts.

## RESULTS

### Ca^2+^ influx of uroepithelial cells induced by bacterial supernatants.

**FIG 1:**
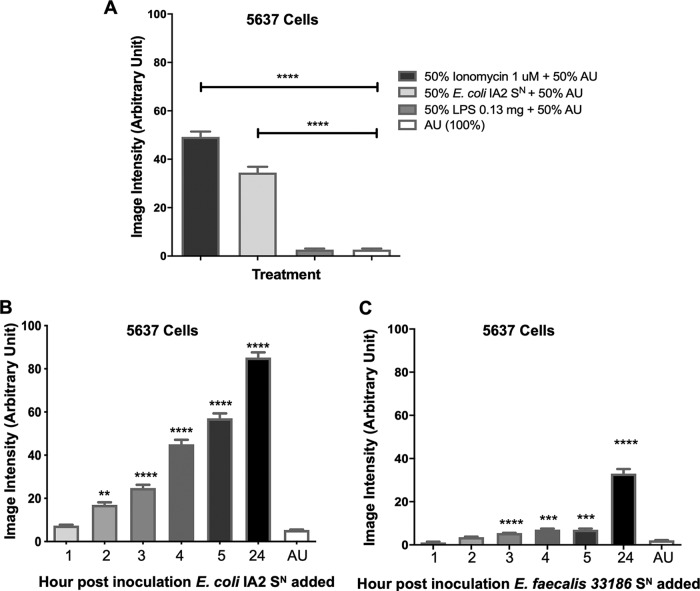
Bacterial supernatant induces Ca^2+^ influx in 5637 uroepithelial cells. Bacterial supernatant (S^N^) of E. coli IA2 after overnight culture was added to 5637 cells at a 50:50 ratio with artificial urine (AU) to assess its ability to induce influx of Ca^2+^ into the cell (A). Supernatants from either E. coli IA2 (B) or E. faecalis 33186 (C) were taken from cultures at 1, 2, 3, 4, 5, and 24 h postinoculum and tested for their ability to induce Ca^2+^ influx in the 5637 cells relative to the AU control. Each bar represents the total average image intensity over 60 s following treatment of a sample. Statistical significance was determined using Dunn’s multiple-comparison test. **, *P* < 0.01; ***, *P* < 0.001; ****, *P* < 0.0001.

In order to determine if bacteria could induce Ca^2+^ influx into uroepithelial cells, bacterial supernatants of the uropathogenic strain Escherichia coli IA2, obtained from an overnight culture, were added to 5637 human urinary bladder cells, and Ca^2+^ influx was measured by fluorescence microscopy. Like ionomycin, the supernatant of E. coli IA2 was able to induce the influx of Ca^2+^ into uroepithelial cells compared to medium alone (artificial urine [AU]) (Fig. 1A). Unlike one previous study (20), lipopolysaccharide (LPS) did not stimulate the influx of Ca^2+^ in this model (Fig. 1A).

Supernatants from E. coli IA2, compared to the noninoculated artificial urine control, increased the levels of Ca^2+^ influx at a significant constant rate from 2 h to the final measurement at 24 h. In contrast, supernatant from Enterococcus faecalis 33186 (another genus implicated in urogenital infections) did not significantly increase the levels of Ca^2+^ influx until the 3-h time point, and the calcium influx appeared to be less than that of the E. coli strain (Fig. 1B and C). These results suggest that uropathogenic bacteria produce and release some excitatory compound that is able to induce Ca^2+^ in uroepithelial cells.

### L. crispatus ATCC 33820 and L. gasseri KE-1 supernatants reduce Ca^2+^ influx caused by E. coli IA2.

**FIG 2:**
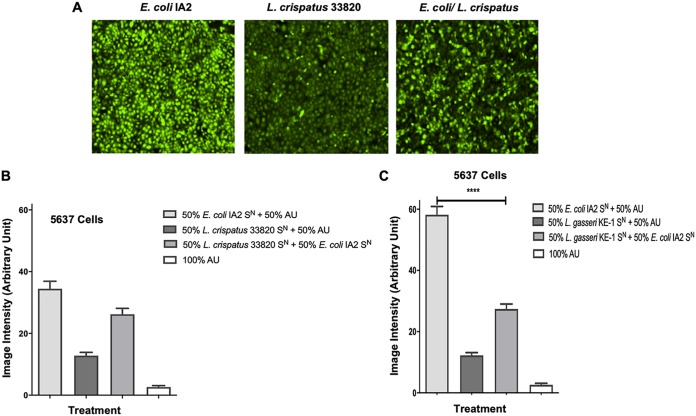
Effect of *Lactobacillus* supernatant on Ca^2+^ influx in 5637 urothelial cells induced by uropathogenic supernatant. (A) Fluorescent microscopy images of Ca^2+^ influx caused by supernatants (S^N^) from E. coli IA2, L. crispatus 33820, and a mixture of supernatants from the two bacteria. Bacterial supernatant from either E. coli IA2, L. crispatus 33820 (B) or L. gasseri KE-1 (C) overnight cultures was mixed 50:50 with either AU or bacterial supernatants to measure Ca^2+^ influx in 5637 urothelial cells (B and C). Each bar represents the total average image intensity over 60 s following treatment of a sample. Statistical significance was determined using Dunn’s multiple-comparison test. ****, *P* < 0.0001.

Given that the uropathogenic bacterial supernatants tested were able to induce Ca^2+^ influx into uroepithelial cells, the next step was to determine what the effect would be if supernatants of healthy commensal urogenital bacteria, such as L. crispatus 33820 and L. gasseri KE-1, were used (21). While the addition of E. coli IA2 supernatant induced high levels of Ca^2+^ influx into the 5637 uroepithelial cells, as seen in Fig. 1, the addition of L. crispatus 33820 supernatant induced very little Ca^2+^ influx (Fig. 2A and B). In addition to this, addition of both the E. coli and L. crispatus supernatants in combination reduced the level of Ca^2+^ influx compared to E. coli alone (Fig. 2A and B). These observations were also seen when supernatants from L. gasseri KE-1 were used, and there was a significant reduction in Ca^2+^ influx into 5637 cells when this was used in combination with the E. coli supernatant (Fig. 2C).

### Extracellular ATP from bacterial supernatants and the ability of L. crispatus to mitigate its effects.

**FIG 3:**
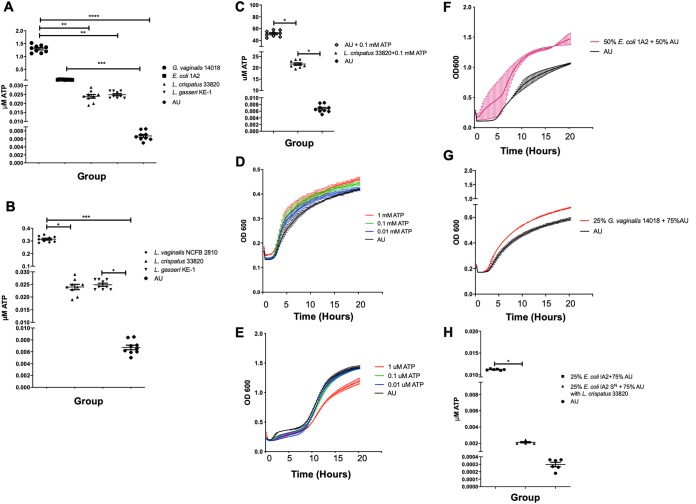

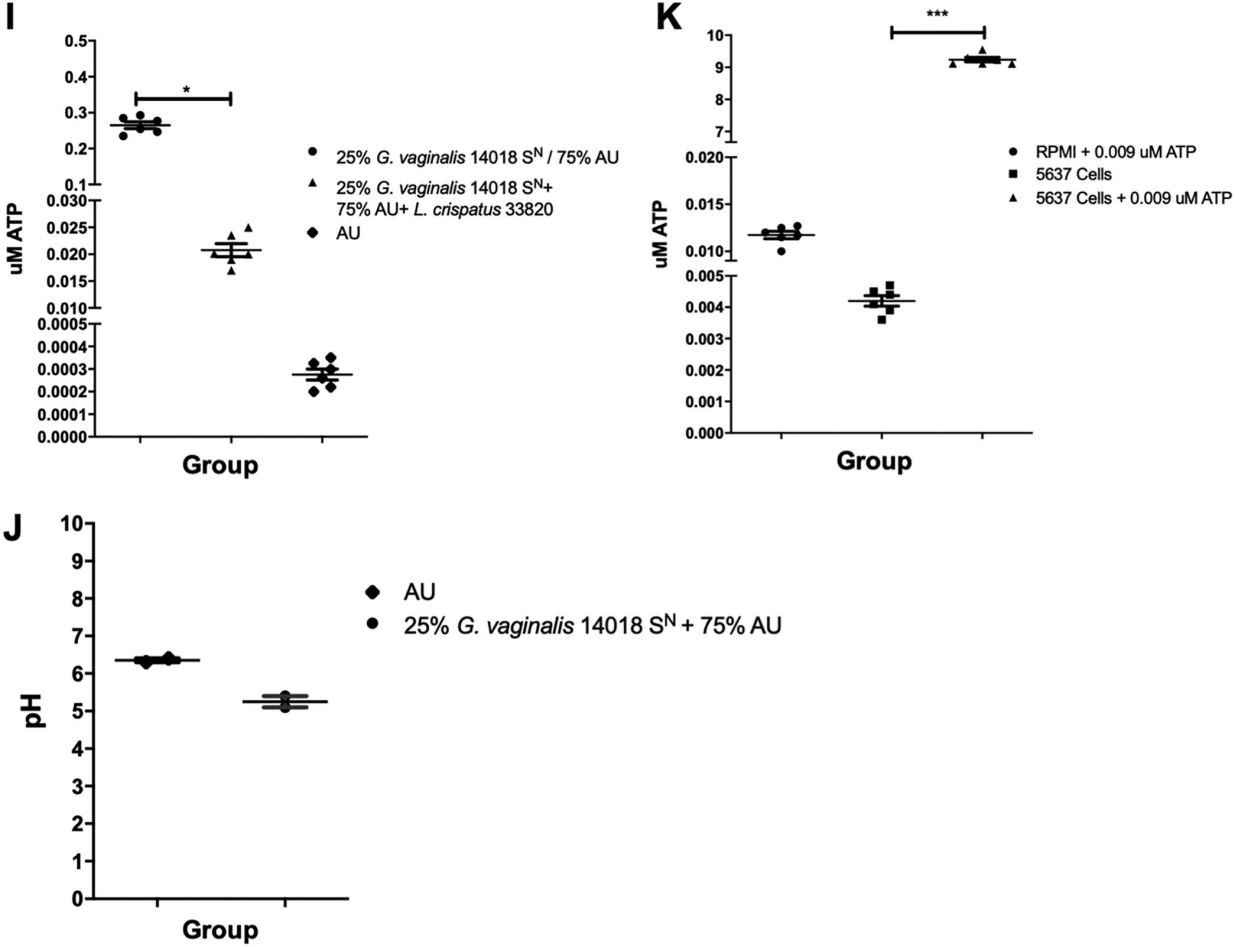
Release and utilization of extracellular and supplemented ATP by bacteria. E. coli, L. crispatus, L. gasseri, *G. vaginalis*, and *L. vaginalis* supernatants (S^N^) were collected from overnight cultures grown in AU and measured for ATP (A and B). Statistical significance was determined using Tukey’s test (*P* ≤ 0.05). L. crispatus was grown in AU supplemented with 0.1 mM ATP overnight, and the amount of ATP was evaluated by luminometer (C). Growth of L. crispatus and E. coli was measured in the presence of different concentrations of ATP in AU (D and E) and additionally for L. crispatus supplemented with E. coli or *G. vaginalis* supernatants (F and G). The ability of L. crispatus to reduce the amount of ATP in AU supplemented with 25% E. coli supernatant (H) and 25% *G. vaginalis* supernatant (I) individually was also examined. L. crispatus 33820 was grown in media supplemented with *G. vaginalis* 14018 to assess the change in pH (J). 5637 urothelial cells were incubated in RPMI, supplemented with small quantities of ATP, and incubated for 2 min to assess the amount of ATP released (K). Statistical significance was determined using Dunn’s multiple-comparison test. *, *P* < 0.05; **, *P* < 0.01; ***, *P* < 0.001; ****, *P* < 0.0001.

There are a number of potential excitatory compounds that could be released by uropathogenic bacteria, such as ATP. In order to determine if, and how much, ATP was being released by these bacteria, a luminescent assay was used to quantify the amount of extracellular ATP released. Supernatants from overnight cultures of E. coli IA2, L. crispatus 33820, and L. gasseri KE-1, as well as Gardnerella vaginalis 14018, an organism commonly found in the reproductive and urogenital tracts and associated with bacterial vaginosis, were tested to determine the concentrations of ATP released. In a separate experiment, *L. vaginalis* NCFB 2810 (another distinctive vaginal commensal) was assessed with L. crispatus, L. gasseri, and artificial urine as comparative controls. Supernatants from the overnight cultures of *G. vaginalis*, *L. vaginalis*, and E. coli contained significantly more ATP than medium alone, while both L. crispatus and L. gasseri produced some ATP (Fig. 3A and B), *L. vaginalis* produced 10-fold more in comparison to the other lactobacilli tested (Fig. 3B). Supernatant from *G. vaginalis* contained significantly more ATP than both L. crispatus and L. gasseri (Fig. 3A).

Given that L. crispatus was able to reduce Ca^2+^ influx and did not release comparatively large amounts of ATP, the utilization of this molecule was assessed. The amount of ATP remaining when L. crispatus was grown in AU supplemented with 0.1 mM ATP for 24 h was less than half that of the control (Fig. 3C). To further characterize ATP reduction by L. crispatus, the bacterium was cultured in AU supplemented with different concentrations of ATP or in AU supplemented with 50% E. coli supernatant and 25% *G. vaginalis* supernatant, which contained bacterially released sources of ATP. The growth of L. crispatus was increased as concentrations of ATP increased (Fig. 3D), while the growth of E. coli was inhibited with increasing concentrations of ATP (Fig. 3E). The growth of L. crispatus was also increased when supplemented with the E. coli (Fig. 3F and H) and *G. vaginalis* (Fig. 3G and I) supernatants. In the presence of ATP or supernatant from *G. vaginalis* that also contained ATP, the pH of L. crispatus became further reduced, indicating its metabolism (Fig. 3J). Direct metabolic use of ATP by lactobacilli has not been demonstrated by this fermentative bacterium to our knowledge.

### Further release of ATP by urothelial cells when stimulated with low concentrations of ATP.

It was unclear whether the concentration of ATP detected in the bacterial supernatants was enough to stimulate cellular Ca^2+^ influx on its own, so we then determined what the effect was when ATP was directly added to 5637 cells. Following stimulation of the 5637 cells in RPMI with 0.009 μM ATP for 2 min, there was a significant increase in the concentration of ATP detected in the cell supernatant compared to the cells-only control (Fig. 3K). This suggested that stimulating uroepithelial cells with ATP is able to induce the release of more ATP into the surrounding environment.

### Effects of subtherapeutic ciprofloxacin on E. coli IA2 on ATP release.

**FIG 4:**
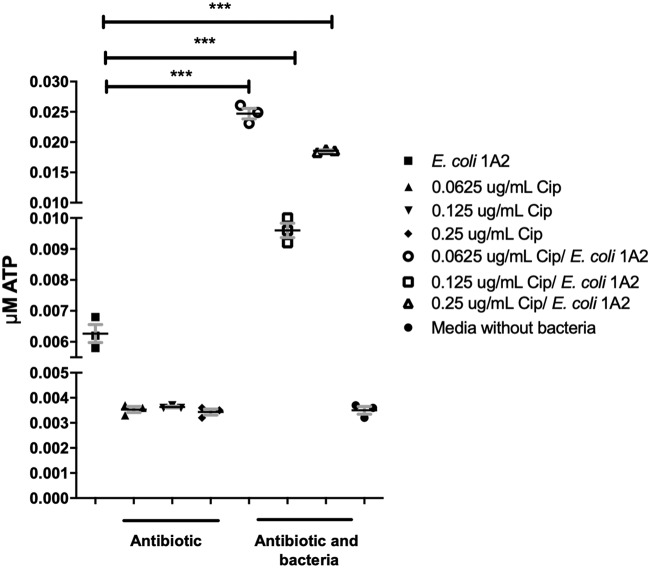
The ability of subtherapeutic concentrations of ciprofloxacin to induce E. coli to release more ATP. Data represent a single biological experiment. This was to determine the minimum inhibitory and subtherapeutic concentrations of exposure to this antibiotic (Cip). E. coli cells were grown in an overnight culture in various sub-MICs of ciprofloxacin and released significant quantities of ATP at different sub-MIC antibiotic concentrations.

Different conditions may influence the ability for uropathogenic bacteria to release ATP. Treatment of bacteria with subtherapeutic concentrations of antibiotics may stress the cells and cause increased ATP release. Preliminary experiments were conducted to determine if subtherapeutic antibiotic treatment altered the ATP release of E. coli IA2. The MIC of ciprofloxacin, an antibiotic routinely used to treat urinary tract infections (UTIs), was determined by culturing the bacteria with concentrations of ciprofloxacin ranging from 10 to 0.031 μg/ml. Under our laboratory conditions, the MIC against E. coli IA2 was determined to be between 1 to 1.5 μg/ml. Next, E. coli IA2 was exposed to sub-MICs of ciprofloxacin in its growth medium at 0.25, 0.125, and 0.0625 μg/ml, respectively. These preliminary experiments showed the treatment with subtherapeutic concentrations of ciprofloxacin caused E. coli IA2 to release more ATP than the cells-only control (Fig. 4).

### Expression of *MAOA* and *MAOB* in the 5637 cells exposed to bacterial supernatants.

**FIG 5:**
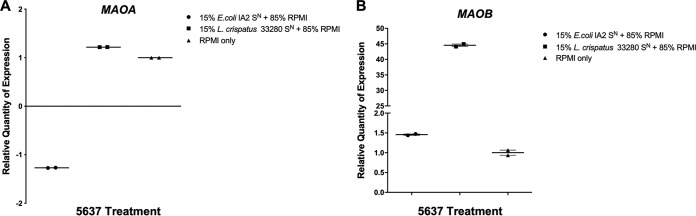
*MAOA* and *MAOB* expression in 5637 urothelial cells following stimulation with bacterial supernatant. Supernatants (S^N^) from overnight cultures E. coli IA2 and L. crispatus 33820 were added to 5637 cell cultures for 3 h, after which cells were lysed and RNA collected. Expression of the genes encoding monamine oxidases (*MAOA*/*MAOB*) was measured by quantitative PCR using *GAPDH* as the reference gene. Samples were normalized to the unstimulated (RPMI) control (A and B). Data are representative of two biological experiments.

Increased influx of intracellular Ca^2+^ caused by excitatory signaling can cause mitochondrial dysfunction. Expression of the genes coding for the mitochondrial enzymes monoamine oxidase A (*MAOA*) and B (*MAOB*) was measured because of their potential ability to degrade neurotransmitters such as serotonin. Bacterial supernatants collected from either E. coli IA2 or L. crispatus 33820 were added to 5637 uroepithelial cells for 3 h, after which the cells were lysed and RNA collected for quantitative PCR (qPCR). Both the E. coli supernatant and the L. crispatus supernatant induced no change in *MAOA* gene expression (Fig. 5A). While the E. coli supernatant had no effect on *MAOB* gene expression, the L. crispatus supernatant increased its expression (Fig. 5B).

### Effect of GABA on Ca^2+^ influx induced by ATP and bacterial supernatant.

**FIG 6:**
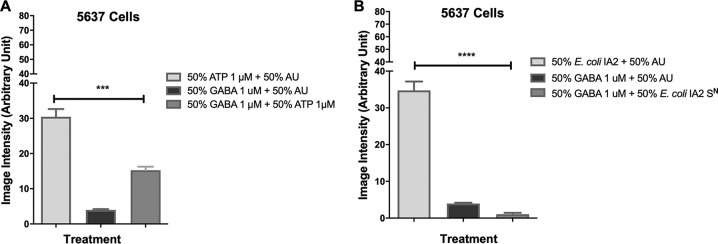
Effect of GABA and ATP on Ca^2+^ influx in 5637 urothelial cells. To evaluate the ability of GABA to inhibit the stimulation of Ca^2+^ influx caused by ATP, AU containing 1 μM GABA was mixed with 1 μM ATP in AU (A). Similarly, to test the ability of GABA to reduce the stimulation of Ca^2+^ influx caused by bacterial supernatant (S^N^), 1 μM GABA was mixed with E. coli IA2 supernatant (B). Statistical significance was determined using Dunn’s multiple-comparison test. ***, *P* < 0.001; ****, *P* < 0.0001.

Given that the Ca^2+^ influx caused by stimulation with ATP is an excitatory nervous system stimulation, it stands to reason that it should be decreased with the addition of an inhibitory signal (22). The neurotransmitter γ-aminobutyric acid (GABA) is the primary inhibitory neurotransmitter in the mammalian central nervous system and is known to inhibit Ca^2+^ influx (20). Therefore, the potential inhibitory effect of GABA against ATP signaling was tested in the uroepithelial cells. The 5637 cells were treated with either 1 μM ATP, 1 μM GABA, or both in combination. Treatment of the 5637 cells with GABA showed reduced Ca^2+^ influx to approximately half the amount compared to the ATP-treated cells alone (30.44 arbitrary units), while influx from the control, GABA in AU, remained low (3.97 arbitrary units) (Fig. 6A). In the following experiment, 5637 cells were treated with bacterial supernatant from E. coli IA2 alone or in combination with GABA, with a control provided by GABA in AU. As previously seen, in the 5637 cells exposed to the E. coli supernatant, there was a high rate of calcium influx (Fig. 6B). Interestingly, the GABA treatment reduced Ca^2+^ influx caused by E. coli supernatant below the levels observed for the control, totally mitigating the effect of the ATP and indicating that some of the interactions may also be combinational between ATP and GABA.

### Effect of bacterial supernatants on myofibroblast contraction.

**FIG 7:**
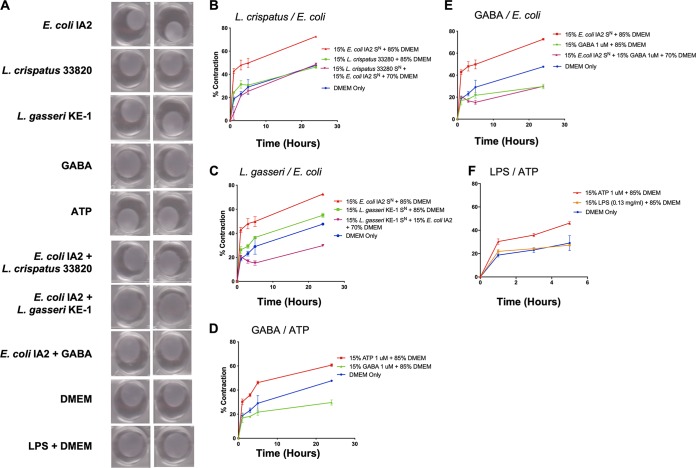
Bacterial supernatants can cause contraction of a myofibroblast-populated collagen matrix. (A) Images of myofibroblast-populated collagen matrix when treated with bacterial supernatants (S^N^) from E. coli IA2, L. crispatus 33820, and L. gasseri KE-1 mixed with DMEM. GABA, ATP, and LPS were included as controls. (B and C) Contraction of myofibroblasts over time when treated with bacterial supernatants from overnight cultures of either E. coli IA2 alone or in combination with L. crispatus 33820 (B) or L. gasseri KE-1 (C) in DMEM. (D and E) Contraction of myofibroblasts when treated with 1 μM ATP or GABA (D) or supernatants from an overnight culture of E. coli IA2 in DMEM (E). (F) Contraction of myofibroblasts when treated with 1 μM ATP or 0.13 mg/ml LPS. DMEM alone was used as a control for all experiments.

In order to determine if Ca^2+^ influx induced by bacterial supernatant or ATP stimulation was sufficient to induce contraction, a collagen contraction assay using primary myofibroblast cells seeded inside a collagen matrix was tested. Myofibroblasts undergo contraction after Ca^2+^ influx, and this *in vitro* model of smooth muscle contraction was used as a surrogate for bladder contractility. The myofibroblasts were cultured in Dulbecco’s modified Eagle’s medium (DMEM) with addition of either supernatants from bacteria, including E. coli IA2, L. crispatus 33820, and L. gasseri KE-1, or GABA and ATP (Fig. 7A). Supernatants from E. coli were able to induce the greatest amount of contraction in the myofibroblasts after 24 h (Fig. 7B), and this was inhibited when the E. coli supernatant was supplemented with supernatant from either L. crispatus (Fig. 7B) or L. gasseri (Fig. 7C).

The addition of ATP induced contraction on myofibroblasts within the first hour, which continued for 24 h (Fig. 7D); while GABA did not cause contraction, it also prevented any basal level of contraction. Addition of GABA to supernatant from E. coli inhibited contraction compared to E. coli supernatant alone to below basal levels with the AU medium only (Fig. 7E). A previous report suggested that the contraction induced by E. coli may be due to LPS stimulation (37). However, after 5 h of exposure to LPS, the myofibroblast contraction was approximately half of that induced by ATP (Fig. 7F).

### Effect of bacterial supernatants on both intracellular α-SMA and induction of TNF.

**FIG 8:**
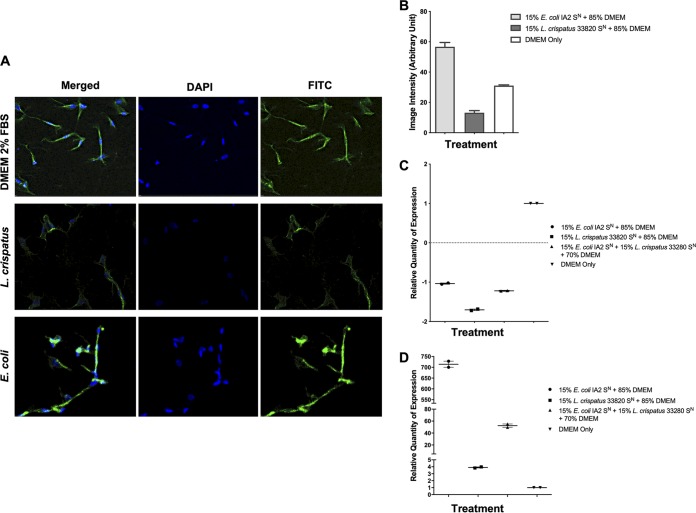
Induction of α-SMA by E. coli IA2 supernatant and mitigation by L. crispatus 33280 supernatant. (A and B) Overnight culture supernatants (S^N^) from E. coli IA2 and L. crispatus 33280 cultures either alone or in combination were cocultured with myofibroblasts for 1 h to determine levels of α-SMA. Image intensity was measured by confocal microscopy with DAPI and fluorescein isothiocyanate (FITC) to show staining of α-SMA. (C and D) Overnight culture supernatants from E. coli IA2 and L. crispatus 33280 cultures either alone or in combination were cocultured with myofibroblasts that were grown in the collagen matrix and then incubated at 37°C with 5% CO_2_ for 3 h, and following this, RNA was extracted. Expression of the genes encoding α-SMA (*ACTA2*) and TNF-α (*TNF*) was measured by quantitative PCR using *GAPDH* as a reference gene. Samples were normalized to the unstimulated control (DMEM) (C and D).

To determine myofibroblast contractive abilities in the presence of bacterial compounds, the effect on alpha smooth muscle actin (α-SMA) was assessed. The α-SMA protein is one of the isoforms of actin and the major constituent of the contractile apparatus. Supernatant from E. coli IA2 was added to myofibroblast cultures, and α-SMA was measured using confocal microscopy after 1 h of exposure. Treatment of myofibroblasts with E. coli supernatant increased the level of α-SMA detected (Fig. 8A and B) compared to the medium-only control. In addition to this, treatment of myofibroblasts with supernatant from L. crispatus 33280 reduced the level of α-SMA compared to the control (Fig. 8A and B). In addition to this, RNA was collected from myofibroblasts following 3 h of exposure in order to determine the expression of the *ACTA2* gene (which encodes α-SMA). Neither the E. coli nor the L. crispatus supernatants appeared to alter gene expression of *ACTA2* (Fig. 8C). Thus, products present in the E. coli supernatant may cause contraction of α-SMA, while supernatant from L. crispatus may inhibit α-SMA without altering gene expression.

Finally, to determine if sustained activation of the Ca^2+^ channel promotes apoptosis by bacterial components in the supernatant, *TNF* (the gene that encodes tumor necrosis factor alpha [TNF-α]) was measured as an indicator. Supernatant from E. coli IA2 caused a large increase in expression of *TNF* (Fig. 8D), whereas exposure to L. crispatus 33820 supernatant resulted in only a small increase. When E. coli and L. crispatus supernatants were combined and added to the cells, the expression of *TNF* was reduced compared to that in the E. coli-only group (Fig. 8D).

## DISCUSSION

The data shown in this study demonstrate that uropathogenic E. coli IA2 can release ATP into artificial urine and cause the influx of Ca^2+^ into 5637 uroepithelial cells (Fig. 2A and 3A). The ability to stimulate the uroepithelium could potentially impact the suburethral space and smooth muscle cells, which may directly affect the contractility of the bladder (23). The *in vitro* myofibroblast model showed that the E. coli supernatant could induce high levels of collagen matrix contraction after 24 h (Fig. 7A and B).

Intracellular Ca^2+^ has many roles inside the cell and regulates important mechanisms such as gene expression, metabolism, and proliferation (24). The release of ATP has been shown previously to be detected extracellularly in E. coli, *Salmonella*, Acinetobacter, *Pseudomonas*, *Klebsiella*, and *Staphylococcus in vitro* (19). In patients with urinary infections, antibiotics are often administered. This reduces the number of bacteria in the lumen, where they are exposed to therapeutic concentrations of the antibiotic. However, bacteria can also be embedded intracellularly in the urothelial cells, where only subtherapeutic concentration of antibiotics may reach (25). The data shown here demonstrate that subtherapeutic exposure to ciprofloxacin can cause E. coli to release higher levels of ATP (Fig. 4), which has the potential to enhance bladder contractility.

The role that the urinary microbiota of incontinent patients may have in uncontrolled voiding is supported by the finding that an abundant member of the microbiota, *G. vaginalis*, releases comparatively large amounts of ATP (Fig. 3A) (14, 15). If these amounts are produced *in vivo*, they may cause urothelial cells to release more ATP in the suburethral space, potentially leading to mitochondrial dysfunction and cell apoptosis.

Commensal bacteria are more abundant than pathogens in the bladder of healthy women and are associated with a reduced risk of UUI (26). This could potentially be occurring by inhibiting the pathogenic bacteria or the pathogenic process. We surmised that they might have a protective role against extracellularly deposited bacterial ATP. This was shown by demonstrating that L. crispatus and L. gasseri did not release significant amounts of ATP (Fig. 3B), and L. crispatus could reduce ATP levels in AU supplemented with 0.1 mM ATP (Fig. 3C). In addition, L. crispatus and L. gasseri inhibited Ca^2+^ influx induced by E. coli*-*derived compounds (Fig. 2A, B, and C). Preliminary evidence was obtained that commensal bacteria could degrade or utilize ATP, with L. crispatus reducing ATP levels in AU. Lactobacillus crispatus also increased expression of the *MAOB* gene (Fig. 5B), encoding proteins that can degrade biogenic amines, which can act as neurotransmitters and include serotonin, dopamine, and many more neuroactive molecules of the class. A decrease in the level of these mitochondrial enzymes has been thought to worsen neurological disorders and may also be another mechanism by which commensal bacteria mitigate the effects of these chemicals (27).

The direct production and then utilization of ATP in media by Gram-negative pathogens was shown by Mempin et al. (19), but the utilization has never been shown for lactobacilli and may provide supplementary ATP. Lactobacilli are typically restricted to glycolytic and fermentative pathways, which produce significantly less ATP than through the respiratory pathways used by other bacteria. If lactobacilli present in the bladder microbiota or even the vagina can scavenge ATP, it may not only potentially provide an extra energy source for the bacteria but could sequester it away from the epithelial layer, thereby promoting a homeostatic environment. These are important findings, since ATP promoted collagen matrix contraction by myofibroblasts (Fig. 7A and D), an *in vitro* model of smooth muscle contraction, suggesting a mechanism for premature voiding and the potential for *Lactobacillus* strains to interfere with this process. However, not all strains of lactobacilli tested were protective against the effects of ATP. Lactobacillus vaginalis, detected in the oral, vaginal, and intestinal microbiomes, has been associated with intermediate grades of bacterial vaginosis (28). In this study, *L. vaginalis* was found to release ATP (Fig. 3B) several fold more than E. coli, which suggests that certain lactobacilli may in fact be part of the disease process; this will require further investigation.

The neurotransmitter GABA is produced by bacteria (29), including certain species of *Lactobacillus*, and this study showed that while it did not cause calcium influx (Fig. 6A) or contraction of myofibroblasts (Fig. 7), it could inhibit contraction caused by E. coli products (Fig. 6B and 7E). Toxins such as hemolysin A from urinary pathogenic Escherichia coli can induce calcium influx and the rapid release of molecules of ATP from erythrocytes and other cells but do not directly involve GABA, which was shown here to mitigate the calcium influx and myofibroblast contraction (30). Further studies that will mitigate the effects of ATP, such as by use of the enzyme apyrase, which catalyzes the hydrolysis of ATP, are planned to determine the relative contribution of bacterial extracellular ATP to pathogenesis. The increase in intracellular Ca^2+^ levels results in the secretion of ATP by urothelial cells (Fig. 3K) by two potential mechanisms. The first mechanism is that ATP can be released via channels such as the connexin hemichannels and pannexin, as well as several anion channels (22). It is possible that stimulation of Ca^2+^ influx in urothelial cells may cause increased expression of vesicular nucleotide transporter (VNUT) in the cell and subsequent release of ATP into the suburethral and muscle layer, causing bladder contraction. The second potential mechanism is that a continuously activated Ca^2+^ channel leads to mitochondrial Ca^2+^ overload, apoptosis, and release of ATP from urothelial cells (31).

Alpha smooth muscle actin (α-SMA) has a well-substantiated, central role in the production of contractile force during wound healing and fibro-constrictive diseases (32). Confocal microscopy demonstrated that there is a direct correlation between increased α-SMA immunoreactivity and uropathogen-induced contraction of the collagen gel matrix by myofibroblasts *in vitro* (Fig. 7B and Fig. 8B and C). There was also a correlation between decreased α-SMA immunoreactivity and a decrease in collagen matrix contraction induced by L. crispatus (Fig. 7B and Fig. 8B and C). However, qPCR showed that these organisms are unable to influence the expression of *ACTA2*. Increased intracellular Ca^2+^ levels can drive the urothelial cells to the apoptosis phase. Tumor necrosis factor alpha can induce apoptosis (33), and so the ability of L. crispatus to reduce the E. coli-stimulated upregulation of this gene in myofibroblast cells could be of significance (Fig. 8D).

In summary, the findings of this study demonstrate a novel mechanism by which uropathogenic bacteria such as E. coli may be able to induce bladder contractility by releasing ATP to trigger Ca^2+^ influx. In addition to this, it shows that commensal members of the urinary microbiota, in particular L. crispatus and L. gasseri, can mitigate the ability of uropathogenic E. coli to stimulate pathways associated with conditions such as UUI. These findings not only provide insight into how bacteria may be able to contribute to disease development, but also identify a potential avenue for treatment using beneficial bacteria. Further studies are required to confirm these mechanisms under *in vivo* conditions.

## MATERIALS AND METHODS

### Bacterial supernatant preparation.

Uropathogenic Escherichia coli 1A2 was maintained on LB agar (Difco, MD), Lactobacillus gasseri KE-1 (urinary isolate), Lactobacillus crispatus ATCC 33820, and Enterococcus faecalis ATCC 33186 were maintained on MRS (deMan, Rogosa, Sharpe) agar (Difco, MD), and Gardnerella vaginalis ATCC 14018 and Lactobacillus vaginalis NCFB 2810 were maintained on Columbia blood agar (CBA) and *Gardnerella* selective agar. For these studies, all strains of bacteria were grown in artificial urine (AU) (34), which in preliminary experiments was shown not to stimulate the influx of Ca^2+^ when in the presence of human cell lines. The recipe for AU was CaCl_2_·H_2_O (0.651 g/liter) MgC1_2_·_6_H_2_O (0.651 g/liter), NaCl (4.60 g/liter), Na_2_SO_4_ (2.30 g/liter), sodium citrate (0.65 g/liter), sodium oxalate (0.02 g/liter), KH_2_PO_4_ (2.80 g/liter), KCl (1.6 g/liter), NH_4_Cl (1.00 g/liter), urea (25.00 g/liter), creatine (1.10 g/liter), and tryptic soy broth (10.00 g/liter), and the pH was adjusted to 5.8. The mixture was sterilized by filtration with a 0.45-pm membrane filter.

Supernatants were collected from bacterial cultures grown overnight (24 h) at 37°C after reaching stationary phase. Cultures were pelleted by centrifugation at 4,500 × *g* (Eppendorf centrifuge 5804 R) for 15 min. The supernatant was pH adjusted to 7.0 with 0.1 M HCl or NaOH, filter sterilized with a 0.22-μm sterile syringe filter, and aliquoted and stored at −20°C until use. In the case of E. coli and E. faecalis, overnight cultures were diluted 1:100 with fresh artificial urine, returned to incubation at 37°C, and sampled at 1, 2, 3, 4, 5, and 24 h for testing. For the experiments involving the addition of supernatants from L. crispatus or L. gasseri to that from uropathogens, the urothelial cells were first treated with L. crispatus or L. gasseri supernatant for 1 min, and then the uropathogenic supernatant was added. In the case of serial dilution, L. crispatus supernatant was diluted 6-fold to the E. coli supernatant.

For investigation of the subtherapeutic concentration of ciprofloxacin, L. crispatus was grown in MRS medium (Difco, MD). Growth curves for these bacteria were generated using a plate reader (Eon Biotek, VT) at the optical density at 600 nm (OD_600_) and 37°C to determine the exponential phase.

### Cell culture.

Bladder epithelial cells (5637 [ATCC HTB-9]) were maintained in RPMI 1640 (Roswell-Park Memorial Institute medium 1640 [Thermo Fisher Scientific, MA]) supplemented with 10% fetal bovine serum (FBS [Thermo Fisher Scientific, MA]) and 2 mM l-glutamine (Thermo Fisher Scientific) at 37°C and 5% CO_2_. The medium was changed every 48 h or more regularly if the cells were confluent (90% to 100%), after washing by 1× PBS and trypsinization by 0.25% trypsin-EDTA (1×) (Gibco), with a ratio of 1 to 10. Primary myofibroblast cells were extracted from the palmar fascia during surgery from normal tissue. Primary cultures were maintained in DMEM with 10% fetal bovine serum (FBS; Life Technologies, Carlsbad, CA, USA), 1% l-glutamine (Life Technologies), and 1% antibiotic-antimycotic solution (Life Technologies) at 37°C in 5% CO_2_. All primary cell lines were used up to a maximum of four passages, after which they were discarded.

### RNA isolation and qPCR from cell lines.

RNA was isolated from the samples (200 ng/μl) using the Ambion by Life Technologies Purelink RNA minikit (Thermo Fisher Scientific, MA), following the manufacturer’s instructions. cDNA was made following the instructions on the Applied Biosystems high-capacity cDNA reverse transcription kit (Thermo-Fisher Scientific, MA), and PCR was conducted using a Master Cycler gradient PCR thermal cycler (Eppendorf, NY). Using *GAPDH* (glyceraldehyde-3-phosphate dehydrogenase) as the housekeeping gene, qPCR was set up with each sample being run on the plate in triplicate for each of the conditions. A list of the primer sequences used can be found in Table S1 in the supplemental material. Power SYBR green PCR master mix was used (Thermo Fisher Scientific, MA).

10.1128/mSphere.00439-19.1 TABLE S1 Quantitative PCR primers used in this study. Download Table S1, PDF file, 0.1 MB. Copyright © 2019 Abbasian et al. 2019 Abbasian et al. This content is distributed under the terms of the Creative Commons Attribution 4.0 International license.

### Fluorescent microscopy of Ca^2+^ influx of 5637 cells.

The influx of Ca^2+^ was measured using the Fluo-4 Direct calcium assay kit (Invitrogen, CA). Samples and reagents were prepared according to the protocol manual provided. Ninety-six-well plates were seeded with 100 μl of 5637 cells at 1 × 10^5^ cells/ml in supplemented RPMI and allowed to reach confluence, which occurred at about 48 to 72 h. Cells were counted by using the Invitrogen Countess automated cell counter (Thermo Fisher Scientific, MA) per the manufacturers’ instructions. Fifty microliters of cell culture medium was removed from the initial 100 μl, and 50 μl of Fluo-4 Direct calcium reagent was added to each well. The plate was incubated at 37°C for 30 min at room temperature while protected from light. Controls included ionomycin (1 μM; Sigma ≥98% high-performance liquid chromatography [HPLC]), ATP (1 μM; Sigma A1852), GABA (1 μM; Sigma BioXtra ≥99%), and LPS (0.13 mg/ml; Sigma L3755). The effect of treatments was assessed using a Nikon epifluorescence Ts2R scope at ×10 magnification at 494 nm for excitation and 516 nm for emission for 60 s. The image intensity was calculated using ImageJ and is indicative of Ca^2+^ influx into the urothelial cell’s cytoplasmic space from either the extracellular environment or intracellular Ca^2+^ stores (from here on referred to as “Ca^2+^ influx”).

### Quantification of ATP.

A luminescent assay kit (BacTiter-Glo microbial cell viability assay; Promega, WI) was used to quantify the amount of extracellular ATP released by the bacteria into the supernatant and released by the cells into the cell media. The Synergy H4 hybrid multimode microplate reader was used to quantify the amount of extracellular ATP.

### Myofibroblast-populated collagen contraction.

A collagen matrix was set up using 1.8 mg/ml sterile collagen and a neutralization solution (35). The neutralization solution was made by mixing Waymouth medium (Sigma, W1625) and 2 parts 0.34 M NaOH (Sigma, 221465). One part neutralization mixture was then added to 4 parts of collagen, mixed with 1 × 10^5^ cells to a final volume of 500 μl, and added to each well in a 24-well plate. After a 45-min incubation at 37°C, 1 ml 2% FBS was added to each well, and the plate was incubated for an additional 72 h at 37°C. The medium was then removed, fresh medium and treatment were added, and the collagen matrix was released using a sterile spatula. The plate was scanned using a Canon PIXMA MP250 immediately after release and also at 1, 3, 5, and 24 h. The size of the collagen matrix was measured using ImageJ, and the percentage of contraction was calculated. To decrease any shock to the myofibroblasts, all bacterial strains were grown in DMEM with 2% FBS.

### Immunocytochemistry.

Myofibroblast cells were cultured in a μ-Slide 8 well (ibidi, 80826) to become fully confluent (90% to 100%). Cells were fixed with paraformaldehyde for 10 min at room temperature and then permeabilized with 0.1% Triton X-100 in PBS. Nonspecific staining was blocked with Background Sniper (Biocare Medical, BS966). Cells were stained by incubation with the monoclonal anti-α-SMA antibody (Sigma, A2547) diluted 1:200 and using Alexa Fluor 488 donkey anti-mouse IgG secondary antibody (ThermoFisher, A-21202) to detect fluorescence. The cells were washed, excess liquid was aspirated, and secondary antibody solution was added (1 to 10 μg/ml) (Alexa Fluor 488 donkey anti-mouse IgG secondary antibody; ThermoFisher, A-21202). 4′,6-Diamidino-2-phenylindole (DAPI) staining was used for nuclei. Confocal images were obtained with a Nikon Eclipse Ti2 (×60 lens objective; Nikon, Canada) and quantified by the methodology of Dössel et al. (36). Fluorescence intensity measurements were obtained from entire cells and analyzed with Image J software. Control specimens were identical to experimental specimens, except they were exposed to an irrelevant isotype-matched antibody.

### Myofibroblast-populated collagen RNA extraction and qPCR.

The myofibroblast-populated collagen RNA extraction protocol used in this study is different from those in earlier studies as it is optimized for higher protein concentrations. After incubation and aspiration of media, the collagen matrix was collected in microcentrifuge tubes for high-speed centrifugation for 5 min, and then the supernatant was discarded. An aliquot of 100 μl prewarmed 0.25 mg/ml collagenase was added to each tube and incubated for 15 min at 37°C. RNA was isolated from the samples using the Direct-zol RNA miniprep kit (Zymo Research, CA) following the manufacturer’s instructions, and TRIzol reagent was used to lyse the samples. The RNA concentration was measured using a NanoDrop ND-1000 (Thermo Scientific). The cDNA was made following the instructions on the Applied Biosystems high-capacity cDNA reverse transcription kit (Thermo-Fisher Scientific), and PCR was conducted using a MasterCycler gradient PCR thermal cycler (Eppendorf, NY). Quantitative PCR was set up with each sample being run on the plate in triplicate for each of the conditions, as described earlier using *GAPDH* as the optimized reference gene. A list of the primers used can be found in Table S1.

### Statistics.

The data are expressed as the mean ± standard error of the mean (SEM). Statistical significance was assessed using one-way analysis of variance (ANOVA) followed by Dunn’s multiple-comparison test (GraphPad Prism 5).
