# Supplementary material for: Potential Role of Extracellular ATP Released by Bacteria in Bladder Infection and Contractility
Source: mSphere. 2019 Sep 4;4(5):e00439-19. doi: 10.1128/mSphere.00439-19 (PMC6731529; doi:10.1128/mSphere.00439-19)
Supplement: TABLE S1 [file mSphere.00439-19-st001.pdf]

Primer	Primer Pair ID	Gene Name	Gene Symbol	Gene ID	Exons
GAPDH Sigma 1235	H_GAPDH_1	Glyceraldehyde-3-phosphate dehydrogenase	GAPDH	2597	9-10
MAOA Sigma 1257	H_MAOA_1	Monoamine oxidase A	MAOA	4128	3-5
MAOB Sigma 1257	H_MAOB_1	Monoamine oxidase B	MAOB	4129	12-13
TNF ThermoFisher Scientific Hs00174128_m1	H-TNF-alpha	Tumor necrosis factor	TNF-α	7124	3-4
ACTA2 ThermoFisher Scientific Hs00426835_g1	H-ACTA2	Actin, alpha 2, smooth muscle, aorta	ACTA2	11475	2-3
